# Acute Generalized Exanthematous Pustulosis in a Setting of Cutaneous Lymphoma

**DOI:** 10.7759/cureus.29754

**Published:** 2022-09-29

**Authors:** Deepa P Budh, Saadiya Hawa, Daniele Rios, Akhila Chilakala, Julian A Paniagua

**Affiliations:** 1 Internal Medicine, St. Barnabas Hospital Health System, Bronx, USA; 2 Epidemiology and Population Health, Stanford University School of Medicine, Stanford, USA

**Keywords:** adverse drug reaction, cutaneous lymphoma, antibiotics, skin reaction, acute generalized exanthematous pustulosis

## Abstract

Acute generalized exanthematous pustulosis (AGEP) is a rare dermatological manifestation of the adverse drug reaction that occurs for a varied duration after the receipt of certain drugs. It manifests as an acute onset of generalized exanthematous pustular reaction with an edematous base. It has a characteristic clinical presentation and rapid resolution soon after the removal of the offending drug. The unique histological finding is that of single-cell necrosis of keratinocytes with edema of papillary dermis accompanied by components of vasculitis and/or exocytosis of eosinophils. Management consists of moist antiseptic dressings, topical steroids, infliximab, the use of systemic steroids if needed, and avoiding antibiotics as much as possible. Here, we present a case of AGEP in a setting of usages of antibiotics like vancomycin, cefepime, and ceftriaxone in a patient with cutaneous lymphoma that resolved after withdrawal of the offending antibiotics.

## Introduction

Acute generalized exanthematous pustulosis (AGEP) is a rare dermatological manifestation of the adverse drug reaction that occurs for a varied duration after the receipt of certain drugs, especially certain antibiotics. It manifests as an acute onset of generalized exanthematous pustular reaction with an edematous base. It usually starts within the folds and spreads to larger surfaces like limbs and the trunk. AGEP has a characteristic clinical presentation. Notable findings on histology include single-cell necrosis of keratinocytes with edema accompanied by components of vasculitis and/or exocytosis of eosinophils. Here, we present a case of AGEP in a patient with cutaneous lymphoma who was exposed to antibiotics like vancomycin, cefepime, and ceftriaxone.

## Case presentation

A 75-year-old female with a past medical history significant for diabetes mellitus, hypertension, coronary artery disease, history of T-cell cutaneous lymphoma in remission, lumbar spinal stenosis secondary to a motor vehicle accident in 2009, status post multiple spinal surgeries, bedridden at baseline presented to the emergency department due to worsening of sacral wounds and knee pain.

**Figure 1:**
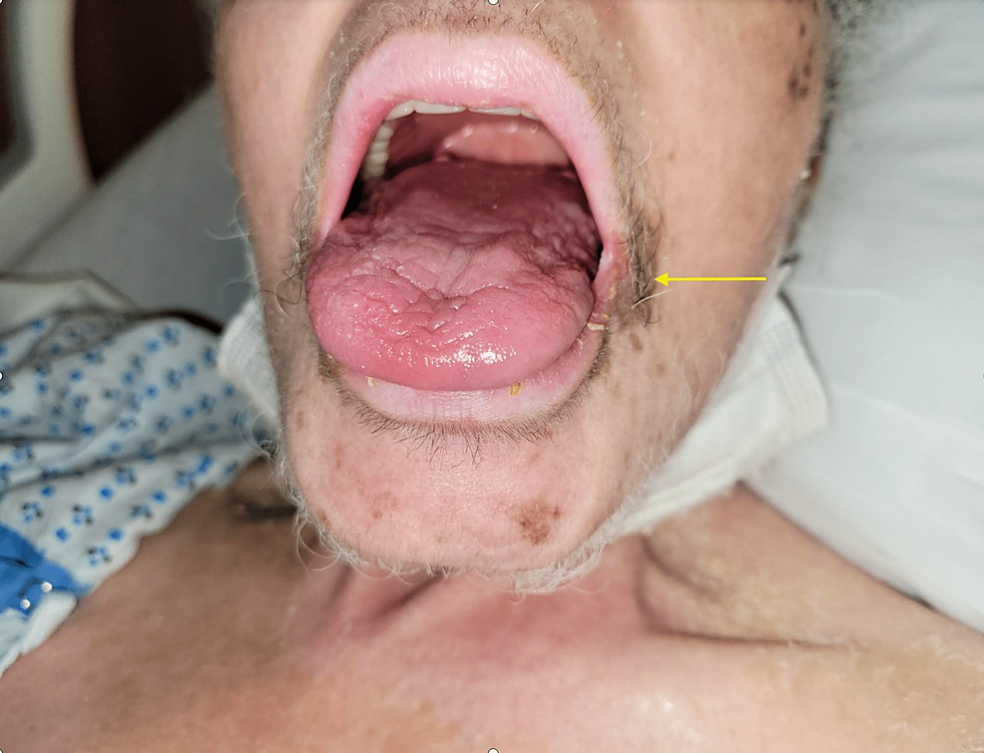
Perioral hyperpigmented patches with multiple pink to violaceous keratotic nodules.

**Figure 2:**
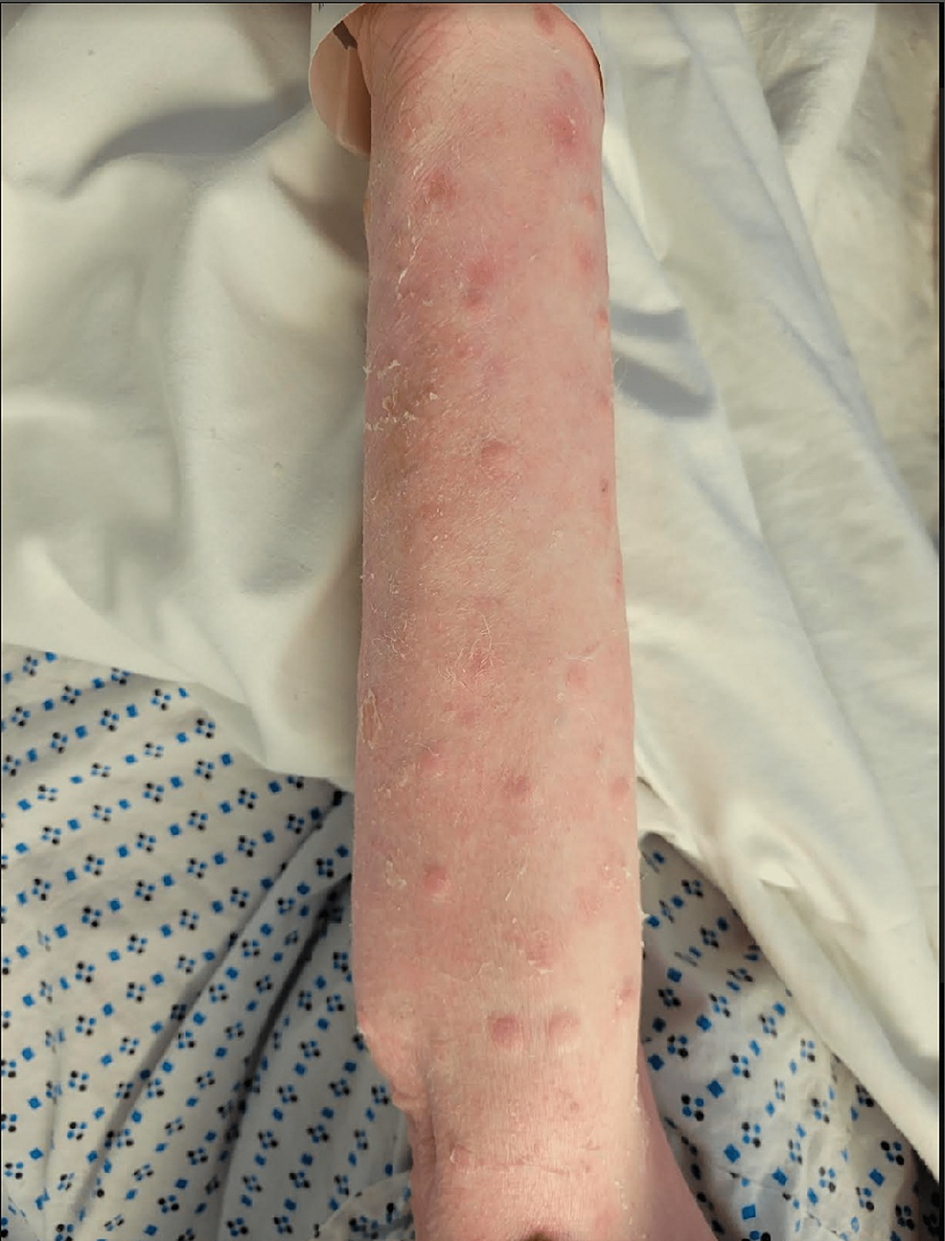
Lower extremity lesions - multiple pink to violaceous keratotic nodules on forearms with generalized skin erythema, and dryness/scaliness.

Upon arrival at the emergency department, the patient was tachycardic and hypotensive. The physical examination revealed four sacral decubitus ulcers, stages 1-4 with the presence of eschar. The patient also had perioral and periorbital hyperpigmented patches, multiple pink to violaceous keratotic nodules on forearms, generalized skin erythema, and dryness/scaliness (Figures 1, 2). The patient stated that she had been recently discharged from another hospital where she was started on multiple medications and presented with rash three days later.

The patient was started on vancomycin and morphine, and laboratory and imaging analysis was obtained. Initial testing was remarkable for leukocytosis (WBC 19,700 uL {reference range 4.2-9.1 10^3^/uL}) with mild left shift and anemia (hemoglobin 9.3 g/dL {reference range 13.7-17.5 g/dL}). The patient was admitted to the general medical floor under the impression of cellulitis of the decubitus infection requiring IV antibiotics. The patient was started on cefepime and metronidazole and continued on vancomycin. Preliminary blood culture results revealed Gram-negative bacilli. The lumbar spine MRI demonstrated a deep sacral decubitus ulcer, without evidence of osteomyelitis. Wound care was consulted and recommended surgical evaluation for wound debridement.

Two days after admission, the patient decompensated and was found to be in a septic state with the development of generalized skin sloughing. She was found to be in a septic state with hypotension, tachycardia, and leukocytosis (WBC: 47,400 × 103/μL {reference range: 4.2-9.1× 103/μL}), and lactic acidemia of 9.2 mmol/L (reference range: 0.5-2.2 mmol/L) along with generalized skin sloughing on bilateral arms, chest, abdomen, and back, and lower mucosal lip desquamation.

**Figure 3:**
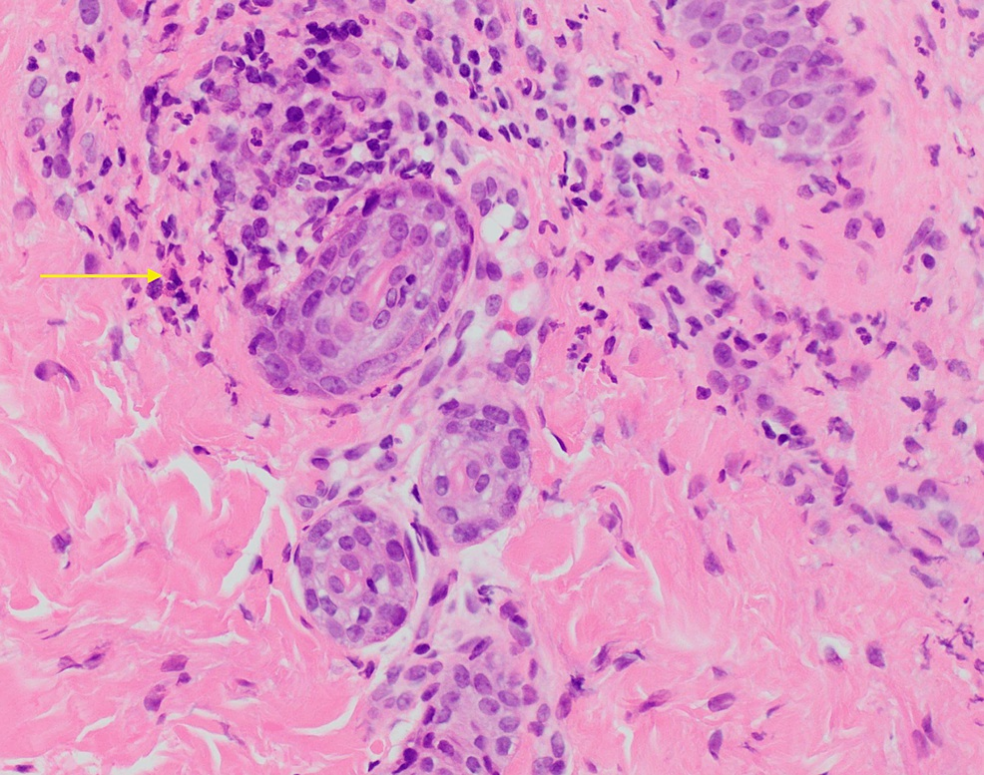
High power view pathology showing aggregates of neutrophils.

**Figure 4:**
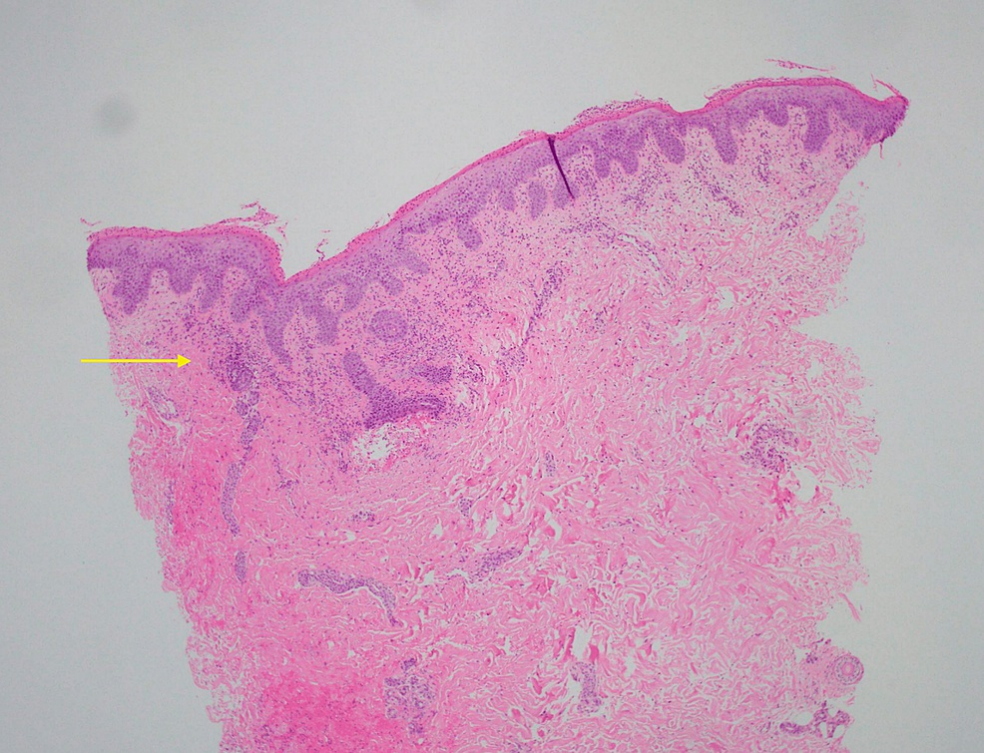
Low power view pathology showing aggregates of neutrophils in the upper dermal layer.

Medical records from a previous admission at another hospital were reviewed and disclosed that the patient had been previously diagnosed with AGEP in the context of being on ceftriaxone given for *Streptococcus mitis* bacteremia three months prior. The antibiotics were switched to meropenem and daptomycin. Blood culture results showed *Enterobacter cloacae* complex, and meropenem was switched to ciprofloxacin. Dermatology was consulted, and due to concerns for Steven Johnson syndrome, the patient was empirically started on intravenous immunoglobulin treatment, while a skin biopsy was arranged. The results from fresh frozen pathology revealed skin with no evidence of apoptotic keratinocytes and/or necrosis; aggregates of neutrophils were seen in the hyperkeratotic layer (Figures 3, 4). The intravenous immunoglobulin was discontinued, and the patient remained on skin emollients.

Progressive improvement with no sloughing was observed after a few days. Her clinical condition improved with regard to the skin lesions. The patient was being treated for the infected decubitus ulcer and discharged in two weeks. However, she succumbed to death three months following this presentation, due to frequent infection of the sacral ulcer while at the nursing home.

## Discussion

The term AGEP was first coined by Beylot et al. in 1980 when referencing drug-induced pustular eruptions with clinical (acute rash in individuals with no history of psoriasis, occurring post-medication use or post-infection) and histological criteria [1]. It is suspected that incidence is one to five cases per million patients every year, but it is believed that many AGEP cases go unreported or incorrectly reported as "drug-induced pustular psoriasis" [2,3].

Drugs, particularly antibiotics, have been noted to cause greater than 90% of reported AGEP cases. Specifically, aminopenicillins, pristinamycin, sulphonamides, quinolones, hydroxychloroquine, terbinafine, and diltiazem are the more frequent causative agents. As the drugs that can cause AGEP vary, the timing of the appearance of the rash can vary based on the high-risk drug, with some rashes appearing 24-48 h later and others 10-14 days later [4].

Acute generalized exanthematous pustulosis presents with pustules on an erythematous edematous base and can be found initially within intertriginous areas and spreads rapidly to larger surface areas, i.e, trunk, and limbs. Generally, the pustules rapidly resolve within a few hours-days post offending drug removal. Histology may indicate “single-cell necrosis of keratinocytes, edema of the papillary dermis, vasculitis, or exocytosis of eosinophils” [4].

AGEP is diagnosed by history, clinical examination, and histology. Based on this the EuroSCAR study group has established standardized scoring criteria (Table 1) [5,6]. The patients are classified as having definite, probable, possible, or no AGEP using this score. There can be some confusion in definitive diagnosis considering the potential overlap of clinical features (Table 2).

**Table 1:** EuroSCAR scoring criteria for acute generalized exanthematous pustulosis. Interpretation - 0: no AGEP; 1-4: possible; 5-7: probable; 8-12: definite; typical: typical morphology; compatible: not typical, but not strongly suggestive of other diseases. AGEP: acute generalized exanthematous pustulosis

Variable	Score
Morphology	
Pustules	
Typical	+2
Compatible	+1
Insufficient	0
Erythema	
Typical	+2
Compatible	+1
Insufficient	0
Distribution	
Typical	+2
Compatible	+1
Insufficient	0
Postpustular desquamation	
Yes	+1
No/insufficient	0
Course	
Mucosal involvement	
Yes	-2
No	0
Acute onset within 10 days	
Yes	0
No	-2
Resolution within 15 days	
Yes	0
No	-4
Fever >38°C	
Yes	+1
No	0
Polymorphonuclear cells >7000/mm^3^	
Yes	+1
No	0
Histology	
Other diseases	-10
Not representative/no histology	0
Exocytosis of Polymorphonuclear cells	+1
Subcorneal and/or intraepidermal non-spongiform or NOS pustules with papillary edema or subcorneal and/or intraepidermal spongiform or NOS pustules without papillary edema (NOS=not otherwise specified)	+2
Spongiform subcorneal and/or intradermal pustules with papillary edema	+3

**Table 2:** Differential diagnoses of acute generalized exanthematous pustulosis. AGEP: acute generalized exanthematous pustulosis; DRESS: drug reaction with eosinophilia and systemic symptoms; SJS: Stevens-Johnson syndrome; TEN: toxic epidermal necrolysis

	AGEP	SJS/TEN	DRESS
Onset	48 h	1-3 weeks	2-6 weeks
Duration	1-2 weeks	1-3 weeks	Many weeks
Clinical findings	Fever, non-follicular pustules	Fever, mucositis	Fever, pustules, exfoliative dermatitis
Lymphadenopathy	+	-	+++
Lab findings	Neutrophilia	Lymphopenia, granulocytopenia	Eosinophilia
Mortality (%)	5	5-35	10

AGEP can be differentiated from pustular psoriasis and other non-follicular pustular diseases like drug reaction with eosinophilia and systemic symptoms (DRESS) and Stevens-Johnson syndrome (SJS) by its acute onset and characteristic morphology and histopathology. The most important differential is pustular psoriasis (PP). PP can be differentiated from AGEP by a slower onset and the presence of a personal or family history. Histologically, the presence of eosinophils and absence of tortuous blood vessels favors AGEP, while the presence of parakeratosis, Munro microabscesses, and tortuous blood vessels favors pustular psoriasis [7,8]. DRESS has an onset of two to six weeks and presents with an erythematous morbilliform rash spreading from the face to the trunk, upper extremities, and lower extremities, rather than pustules, and with mucosal involvement [9]. Stevens-Johnson syndrome/toxic epidermal necrolysis (SJS/TEN) presents with epidermal sloughing and mucosal involvement. A positive Nikolsky sign is characteristic. Histologically, TEN involves full-thickness necrosis of the epidermis along with lymphocytic infiltrates at the dermo-epidermal junction.

Withdrawal of the suspected offending drug should be the first step in the management. It usually results in complete resolution [5]. Moist antiseptic dressings can be used. Antibiotics are avoided unless there is evidence of infection. Topical steroids are sometimes recommended [10]. For selected cases therapies like oral corticosteroids, and infliximab have proven useful [11].

## Conclusions

AGEP is a rare disorder, commonly confused with the diagnosis of TEN, SJS, and DRESS. However, the typical onset time, pattern of distribution, and resolution after discontinuation of the offending agents (antibiotics) are important for the clinical diagnosis as well as management. The use of antibiotics to treat infections in a patient with a history of cutaneous lymphoma creates a diagnostic dilemma. However, clinical manifestations, histology findings, and identifying offending agents are potential requirements for the management of a case of AGEP. Keen clinical observation with timely multidisciplinary involvement leads to a better prognosis and patient care.
